# Maritime Infrared Small Target Detection Based on the Appearance Stable Isotropy Measure in Heavy Sea Clutter Environments

**DOI:** 10.3390/s23249838

**Published:** 2023-12-15

**Authors:** Fan Wang, Weixian Qian, Ye Qian, Chao Ma, He Zhang, Jiajie Wang, Minjie Wan, Kan Ren

**Affiliations:** 1 School of Electronic and Optical Engineering, Nanjing University of Science and Technology, Nanjing 210094, China; wangfan@njust.edu.cn (F.W.);; 2 Jiangsu Key Laboratory of Spectral Imaging and Intelligent Sense, Nanjing University of Science and Technology, Nanjing 210094, China

**Keywords:** infrared small target, gradient histogram equalization measure (GHEM), local optical flow consistency measure (LOFCM), appearance stable isotropy measure (ASIM)

## Abstract

Infrared small target detection plays a crucial role in maritime security. However, detecting small targets within heavy sea clutter environments remains challenging. Existing methods often fail to deliver satisfactory performance in the presence of substantial clutter interference. This paper analyzes the spatial–temporal appearance characteristics of small targets and sea clutter. Based on this analysis, we propose a novel detection method based on the appearance stable isotropy measure (ASIM). First, the original images are processed using the Top-Hat transformation to obtain the salient regions. Next, a preliminary threshold operation is employed to extract the candidate targets from these salient regions, forming a candidate target array image. Third, to distinguish between small targets and sea clutter, we introduce two characteristics: the gradient histogram equalization measure (GHEM) and the local optical flow consistency measure (LOFCM). GHEM evaluates the isotropy of the candidate targets by examining their gradient histogram equalization, while LOFCM assesses their appearance stability based on local optical flow consistency. To effectively combine the complementary information provided by GHEM and LOFCM, we propose ASIM as a fusion characteristic, which can effectively enhance the real target. Finally, a threshold operation is applied to determine the final targets. Experimental results demonstrate that our proposed method exhibits superior comprehensive performance compared to baseline methods.

## 1. Introduction

The Infrared (IR) Search and Track (IRST) system is widely applied in maritime patrols, border surveillance, and maritime rescue operations, making it a critical device in the field of maritime security. IR small target detection is a key technology of the IRST system [1]. The timely detection and localization of small targets on the sea surface, such as distant ships, are essential for mission success and platform safety. Therefore, IR small target detection technology holds significant relevance to maritime security, attracting extensive research efforts. Researchers face common challenges in most scenarios. For instance, small targets occupy only a limited number of pixels, lacking distinct contour and texture features. Numerous detection methods have been proposed to address these difficulties and achieved good outcomes.

However, new challenges arise when heavy clutter, such as waves, sun glints, and island edges, is present on the sea surface. Sea clutter can cause dramatic fluctuations in the background, leading to reduced local contrast of small targets and potential missed detections [2]. Additionally, certain sea clutter exhibits high brightness and shares similar appearance characteristics with small targets, making it challenging to suppress and resulting in serious false alarms. Existing algorithms do not perform well when faced with these issues. As a result, maritime IR small target detection in heavily cluttered environments remains challenging and requires further in-depth research.

### 1.1. Related Work

According to the basic principle, the existing detection methods can be divided into four categories: methods based on background subtraction, methods based on the human visual system(HVS), methods based on optimization, and methods based on deep learning.

The first category is the methods based on background subtraction, which predicts the background image and then subtract the background from the original image to obtain the target image. Classical background subtraction methods include the Top-Hat transformation [3], the Max-mean/Max-median filtering [4], the two-dimensional least mean square filtering (TDLMS) [5], and the Low-pass filter (LPF). Many modifications to the classic methods have been proposed and are widely applied. The histogram rightwards cyclic shift binarization (HRCSB) combines background subtraction with histogram curve transformation [6]. The new white top-hat (NWTH) transformation improves the detection performance by structure element construction and operation reorganization [7]. Double-layer two dimensional least mean square filter uses different filter settings for background suppression and target enhancement [8]. These methods assume that the background is flat. They work well in simple backgrounds, but they cannot cope with complex backgrounds because the heterogeneous regions in complex backgrounds will bring difficulties to background prediction.

The second category is the methods based on target enhancement, which can directly suppress the background and enhance the target by filtering. The difference of Gaussian (DoG) [9] and the Laplacian of Gaussian (LoG) [10] are two classic methods considering the Gaussian model of small targets’ gray distribution. Inspired by the human visual system (HVS) mechanism, researchers developed many detection methods using the local contrast or gray difference [11]. The calculation of these methods usually takes the form of center-surround difference. For example, the average absolute gray difference (AAGD) is a typical center-surround difference method, but AAGD does not utilize directional information, so it cannot suppress clutter edges [12]. The absolute average difference with cumulative directional derivatives (AADCDD) improved AAGD by introducing direction information [13]. Absolute directional mean difference (ADMD) also used direction information to suppress structural backgrounds [14]. The local contrast measure (LCM) proposed a classic nine-cell sliding window [15]. Currently, many improvements in LCM have been developed. For example, the improved LCM (ILCM) [16] and the novel LCM (NLCM) [17] divided the image into many sub-blocks to reduce computation. The multi-scale patch-based contrast measure (MPCM) adopted the product of the differences in opposite directions [18]. The tri-layer LCM (TLLCM) adopted a tri-layer nested window [19]. The homogeneity-weighted LCM (HWLCM) combined the LCM with the homogeneity of the cells [20]. The weighted strengthened local contrast measure (WSLCM) combined the strengthened LCM and the weighting function [21]. The above methods belong to the spatial filtering methods, which are easy to implement and fast to calculate, but they did not take advantage of the target’s motion information. Considering the gray fluctuation caused by the target motion in the time domain, researchers developed many spatial–temporal filtering methods. The spatial–temporal local contrast filter (STLCF) [22] and the spatial–temporal local contrast map (STLCM) [23] calculated the temporal and spatial local contrast of moving small targets separately and then fused them by multiplication. The spatial–temporal local difference measure (STLDM) directly detected targets in the 3-D spatial–temporal domain [24]. The novel spatiotemporal saliency method (NSTSM) is proposed for LSS IR target detection, which utilizes the variance characteristics and gray intensity characteristics of target pixels and background pixels in the spatiotemporal domain [25]. These methods based on target enhancement can effectively suppress the heterogeneous regions in complex backgrounds, but they cannot deal with the clutter similar to the real small target.

The third category is the methods based on optimization, which transforms the small target detection problem into an optimization problem of recovering sparse and low-rank matrices. The IR patch-image (IPI) model assumed that the background matrix is low rank and the target matrix is sparse, and then restores the target image by optimization [26]. Then, this model was extensively researched, and many researchers have proposed improved methods based on the IPI model. Dai et al. proposed the weighted IR patch-image (WIPI) model to solve the problem of excessive target shrinkage, in which the target likelihood coefficient is designed as the weight of the target patch-image [27]. Dai et al. also proposed the non-negative IR patch-image model based on partial sum minimization of singular values (NIPPS) to suppress strong edges better, which replaces the kernel norm in the IPI model with the partial sum of singular values [28]. Like NIPPS, Zhao et al. also tried to improve the sparse term in the IPI model and proposed the method based on non-convex optimization with Lp-norm constraint (NOLC), which replaces the nuclear norm with the Lp-norm [29]. Considering that the single subspace assumption is not suitable for the estimation of complex backgrounds, many researchers proposed models based on multiple subspaces. He et al. proposed the low-rank and sparse representation (LRSR) model, which constructs over-complete dictionaries for sparse representation of small targets [30]. Wang et al. proposed the stable multi-subspace learning (SMSL), which adopts the subspace learning strategy to improve the ability to resist complex background and noise [31]. To make the most of prior information, Dai et al. extended the IPI model to the tensor field and proposed the IR patch-tensor (IPT) model [32]. Then, a large number of methods based on the IPT model began to appear. The partial sum of tensor nuclear norm (PSTNN) can achieve very fast computing speed [33]. The non-convex tensor rank surrogate joint local contrast energy (NTRS) utilized a non-convex tensor rank surrogate merging tensor nuclear norm and the Laplace function for background patch constraint [34]. Many researchers have extended the tensor model to the spatial–temporal domain, such as the multiple subspace learning and spatial–temporal IPT (MSL-STIPT [35]), the spatial–temporal tensor model (STTM) [36], and the novel spatial–temporal tensor model with saliency filter regularization (STTM-SFR) [37]. These optimization-based methods can continuously improve performance through model refinement. However, their assumption of low-rank background makes them highly demanding of background uniformity, and some clutter also confirms the assumption of local sparse. Meanwhile, they are generally time-consuming due to iterative operations.

The fourth category is the methods based on deep learning, which first trains the model to mine image features using a large number of data sets and then detect small targets using the trained model. Convolutional neural network (CNN) is a commonly used network that is beneficial to learning infrared image hierarchical features. Zhao et al. proposed the novel lightweight convolutional neural network called the TBC-Net, which consists of two modules: target extraction and semantic constraints [38]. With these two modules, TBC-Net can add high-level semantic constraint information on images into training. Since pooling layers in CNN can cause the targets in deep layers to be missed, Li et al. proposed a dense nested attention network (DNA-Net), which can preserve the target deep features through progressive interaction between the high-level and low-level features [39]. Considering that CNN cannot capture large-scale dependencies, many researchers have begun to use transformer based on self-attention mechanisms. Liu et al. proposed an IR small-dim target detection method with the transformer, which uses a feature enhancement module to improve feature learning for small-dim targets [40]. Chen et al. proposed a hierarchical vision transformer-based method called the IRSTFormer, specifically used for small target detection in large-size images [41]. Due to the small size and few features of IR small targets, it is difficult for pure data-driven methods to achieve high performance. Researchers have tried to combine neural networks with traditional small-target models. Dai et al. proposed a novel model-driven deep network, which introduces the local contrast and extends the application of traditional small target features in the field of neural networks [42].

The performance of deep learning-based methods relies heavily on a large number of training samples. However, obtaining an ample amount of training samples for IR small target detection is challenging in military scenarios, which hinders the application of deep learning approaches. Therefore, there is a need for further development of models based on small samples.

While the aforementioned existing methods demonstrate good performance in many scenarios, they struggle to handle heavy sea clutter environments. Extensive clutter on the sea surface can significantly interfere with the detection process. Some clutter may exhibit a very similar appearance to real small targets, posing difficulties for existing methods to suppress them effectively.

### 1.2. Motivation

**Figure 1:**
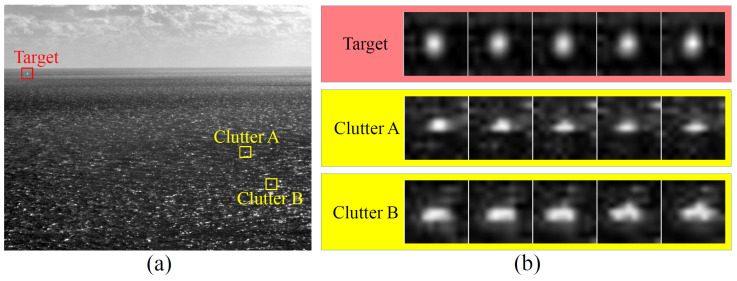
Schematic diagram of an IR image and typical salient regions. (**a**) An IR image with a small target and heavy sea clutter. (**b**) Local images of the marked regions within five consecutive frames.

At a long imaging distance, the projection of a small target on the camera focal plane typically covers only a few pixels or even less than one pixel. However, due to scattering, diffraction, and focusing effects, the emitted IR radiation from the target undergoes diffusion, resulting in an image spot that is larger than the target’s physical size. Although this spot cannot be perfectly circular, it approximates isotropy to some extent. The IR radiation of the target and the properties of the optical system will not change dramatically, allowing for the appearance of the target spot to remain relatively stable over a short period of time. Figure 1a illustrates an IR image containing a small target and heavy sea clutter, with the small target marked by a red box. The first line of Figure 1b shows the local images of the small target captured in five consecutive frames. It can be observed that the appearance of the small target exhibits little variation over these five frames and consistently maintains an approximate isotropic appearance. The combination of isotropy in the spatial domain and stability in the temporal domain is referred to as the Appearance Stable Isotropy.

Sea clutter primarily consists of waves and sun glints. The IR radiation emitted by the waves gives rise to heterogeneous regions in the background, with its appearance being influenced by the shape of the waves. Sun glints, on the other hand, are bright spots formed due to sunlight reflecting off the sea surface, and their appearance depends on local reflective surfaces. The sea surface itself exhibits significant randomness, resulting in irregular shapes and varying sizes of clutter. In an image, both isotropic and anisotropic clutter can coexist simultaneously. Furthermore, the dynamic nature of the sea surface leads to continuous deformation of clutter. An initially isotropic clutter can rapidly transform into an anisotropic one within a short duration. Consequently, sea clutter does not maintain a consistent appearance and lacks the characteristic of Appearance Stable Isotropy.

In Figure 1a, two examples of clutter are marked with yellow boxes, labeled as Clutter A and Clutter B, respectively. The second and third lines of Figure 1b display the local images of Clutter A and B, respectively, captured over five consecutive frames. It can be observed that Clutter A initially appears isotropic in the first frame but undergoes subsequent changes in shape. By the fifth frame, Clutter A has become significantly anisotropic. On the other hand, Clutter B consistently demonstrates anisotropy.

The above analysis and examples demonstrate substantial differences between small targets and sea clutter in terms of ASI. Leveraging this distinction can effectively differentiate between them.

Based on the above analysis, this paper proposes a detection method that utilizes the appearance stable isotropy measure (ASIM). The contributions of this paper can be summarized as follows:

(1) The Gradient Histogram Equalization Measure (GHEM) is proposed to effectively characterize the spatial isotropy of local regions. It aids in distinguishing small targets from anisotropic clutter.
(2) The Local Optical Flow Consistency Measure (LOFCM) is proposed to assess the temporal stability of local regions. It facilitates the differentiation of small targets from isotropic clutter.
(3) By combining GHEM, LOFCM, and Top-Hat, ASIM is developed as a comprehensive characteristic for distinguishing between small targets and different types of sea clutter. We also construct an algorithm based on ASIM for IR small target detection in heavy sea clutter environments.
(4) Experimental results validate the superior performance of the proposed method compared to the baseline methods in heavy sea clutter environments.

The remainder of this paper is organized as follows: Section 2 presents the proposed method, detailing its key components. Subsequently, in Section 3, comprehensive experimental results and analysis are provided. Finally, this paper is concluded in Section 4.

## 2. Proposed Method

**Figure 2:**
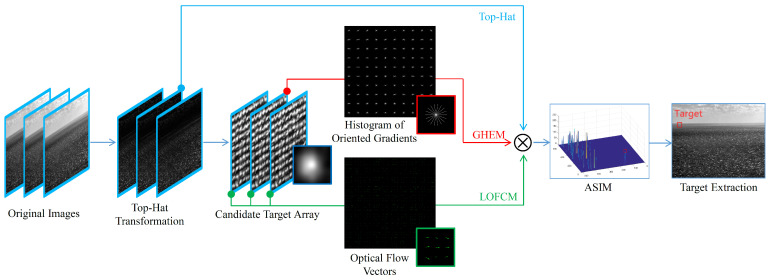
Flowchart of the proposed method.

Figure 2 shows the flowchart of the proposed method. Firstly, the original images are processed using the Top-Hat transformation to obtain the salient regions. Then, a preliminary threshold operation is performed to extract candidate targets from the salient regions. The characteristic neighborhoods of these candidate targets are then scaled and stitched to form a candidate target array. Third, the oriented gradient histogram of the candidate targets is computed, and GHEM is used to characterize their isotropy. At the same time, the local optical flow vectors of the candidate targets are calculated, and LOFCM is used to characterize their appearance stability. Fourth, ASIM is obtained by fusing GHEM, LOFCM, and Top-Hat image. ASIM serves as an effective comprehensive feature for distinguishing between small targets and various types of sea clutter. Finally, by applying a threshold operation on ASIM, the true target can be extracted.

### 2.1. Candidate Target Extraction

Both small targets and sea clutter are salient regions in IR images, characterized by their higher brightness than the surrounding background. In this paper, the Top-Hat transformation is utilized to extract these salient regions as candidate targets. As a background subtraction method, the Top-Hat transformation can effectively preserve the grayscale distribution of the salient areas, facilitating the subsequent description of their appearance features.

The Top-Hat transformation is defined as:

(1) T(I)=I−(I∘S),

(2) I∘S=(I⊖S)⊕S,

where *I* is the original image; T(I) is the Top-Hat image; *S* is the structure element; ∘ represents the morphology open operation; and ⊕ and ⊖ represent expansion and corrosion, respectively.

**Figure 3:**
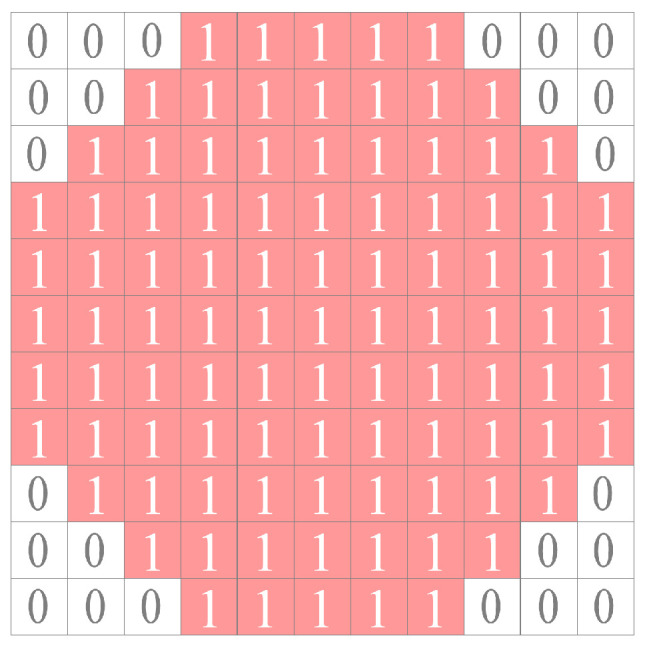
Structure element. The value of the effective pixels are 1, marked in red. The value of the invalid pixels are 0, marked in white.

The size of the structure element should be adaptable to the salient regions [7]. If the structure element is too small to cover the candidate targets, it may result in the loss of some target pixels. Conversely, if the structure element is too large, background suppression will be incomplete, increasing the difficulty of subsequent processing. The ideal structure element should be slightly larger than the candidate targets. According to the definition provided by the Society of Photo-Optical Instrumentation Engineers (SPIE), small targets typically range in size from 2 × 2 to 9 × 9 [15]. Although sea clutter has a wider range of sizes, only small ones can easily be mistaken for small targets. Large-sized clutter is easy to suppress since it responds poorly to small target detection algorithms. Therefore, in this paper, the size of the structure element is set to 11 × 11, slightly larger than the 9 × 9 size of small targets, as shown in Figure 3.

**Figure 4:**
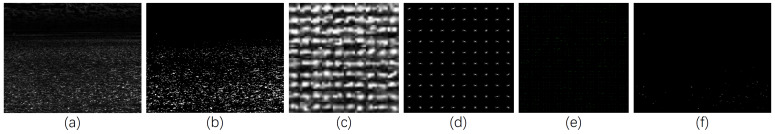
Intermediate results of the proposed method. (a) Top-Hat image. (b) Binary image. (c) Candidate target array. (d) Visual HOG of the candidate target array. (e) Optical flow vectors of candidate target array. (f) ASIM image.

Figure 4a shows the Top-Hat image of Figure 1a. In Figure 4a, homogeneous backgrounds are suppressed while salient regions are retained. Then, the salient regions are extracted using a preliminary threshold segmentation.

(3) $$TH_{\mathrm{Top}-\mathrm{Hat}}=\mu_{\mathrm{Top}-\mathrm{Hat}}+k_{\mathrm{Top}-\mathrm{Hat}}\times \sigma_{\mathrm{Top}-\mathrm{Hat}}$$

where $TH_{\mathrm{Top}-\mathrm{Hat}}$ is the threshold; $\mu_{\mathrm{Top}-\mathrm{Hat}}$ and $\sigma_{\mathrm{Top}-\mathrm{Hat}}$ are the mean and standard deviation of the Top-Hat image, respectively; $k_{\mathrm{Top}-\mathrm{Hat}}$ is the coefficient. Since the brightness of the target may be lower than many sea clutter, the threshold can be set to a small value to ensure that the target can be retained. In this paper, $k_{\mathrm{Top}-\mathrm{Hat}}$ is recommended to be set to 1 according to development experience. The binary image BW can be obtained using the preliminary threshold segmentation.

(4) $$\mathrm{BW}=\left\{\begin{array}{cc} 1 & T\geq TH_{\mathrm{Top}-\mathrm{Hat}}\\ 0 & T<TH_{\mathrm{Top}-\mathrm{Hat}} \end{array}\right.$$

The binary image is shown in Figure 4b. There are many connected domains in Figure 4b, each representing a salient region. These salient regions include small targets and sea clutter. According to the analysis of the small target size mentioned above, only connected domains with areas ranging from 2 × 2 to 9 × 9 are considered candidate targets, while the others are disregarded. The coordinates of the candidate target are represented by the centroid of the connected domain.

(5) $$\left(x,y\right)=\left(\frac{\sum_{i=1}^{np}x_{i}\times T\left(x_{i},y_{i}\right)}{\sum_{i=1}^{np}T(x_{i},y_{i})},\frac{\sum_{i=1}^{np}y_{i}\times T\left(x_{i},y_{i}\right)}{\sum_{i=1}^{np}T(x_{i},y_{i})}\right)$$

where (x,y) is the coordinates of the centroid; np and $\left(x_{i},y_{i}\right)$ are the number and coordinates of the pixels in the connected domain, respectively.

Assuming the presence of *N* candidate targets, their sizes randomly range from 2 × 2 to 9 × 9, which is still a wide range. When candidate targets possess too few pixels, it can lead to inaccurate calculations of appearance features. Hence, it is necessary to scale the candidate targets in order to adjust their size appropriately. The region within the minimum enclosing square of the connected domain is defined as the characteristic neighborhood of the candidate target, which will be subsequently scaled to 11 × 11 using bilinear interpolation. The equal aspect ratio scaling ensures that the appearance feature of the candidate targets remains unchanged. If the candidate target is 9 × 9, its minimum enclosing square would coincidentally be 11 × 11, and no further scaling is needed.

After the scaling is completed, all candidate targets are sorted based on their maximum values in the Top-Hat image. The characteristic neighborhoods of these candidate targets are then concatenated into a square image named the candidate target array image. The concatenation is performed in a top-to-bottom and left-to-right order. If the number of candidate targets is not a perfect square, any empty positions in the array will be filled with zero matrices. Figure 4c illustrates the candidate target array image extracted from Figure 4b. Additionally, a position mapping function is employed to record the correspondence between the positions in the candidate target array image and the original image.

(6) $$P_{original}=M\left(P_{array}\right)$$

### 2.2. Gradient Histogram Equalization Measure (GHEM)

The histogram of oriented gradients (HOG) is a popular feature descriptor extensively employed in computer vision for target detection and recognition tasks. HOG describes the appearance features of local regions by quantifying the distribution of pixel gradients and orientations. In this paper, the HOG technique is utilized to analyze the appearance features of candidate targets. Specifically, if a candidate target exhibits isotropy, the gradients within its characteristic neighborhood should be evenly distributed across different directions. In contrast, anisotropic candidate targets will have gradients concentrated in certain directions. Consequently, the gradient histogram equalization measure (GHEM) is proposed to measure the isotropy level of candidate targets.

The gradient and orientation of the candidate target array are calculated as follows:

(7) $$A_{x}\left(x,y\right)=A\left(x,y\right)-A\left(x-1,y\right),$$

(8) $$A_{y}\left(x,y\right)=A\left(x,y\right)-A\left(x,y-1\right),$$

(9) $$G\left(x,y\right)=\sqrt{A_{x}^{2}\left(x,y\right)+A_{y}^{2}\left(x,y\right)},$$

(10) $$\theta \left(x,y\right)=\arctan\left(\frac{A_{y}\left(x,y\right)}{A_{x}\left(x,y\right)}\right),$$

where A(x,y) is the pixel value in the candidate target array image; $A_{x}$ and $A_{y}$ are horizontal and vertical gradients, respectively; *G* is the magnitude of the gradient; θ is the gradient direction; and its range is $\left[0^{\circ },\;180^{\circ }\right]$ after taking the absolute value.

**Figure 5:**
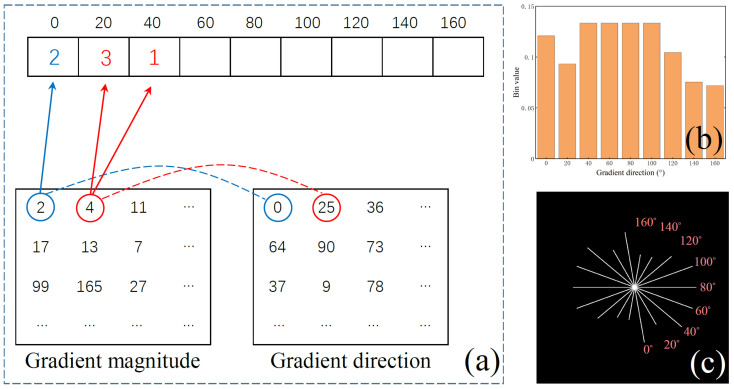
Related schematic diagrams of HOG. (a) Schematic diagram of HOG calculation. The two pixels marked with blue and red circles are used as examples. (b) A calculation result of HOG. (c) Schematic diagram of visual HOG.

Next, Figure 5a shows the process of constructing the oriented gradient histogram. The range of $\left[0^{\circ },\;180^{\circ }\right]$ is divided equally into nine parts, each covering a range of 20 degrees. As a result, an empty histogram with nine bins is created to accommodate the distribution of gradient values. Within the characteristic neighborhood, each pixel has a gradient magnitude value and a corresponding gradient direction. The gradient value will be assigned to the corresponding bin based on the corresponding direction value. For example, in Figure 5a, the pixel marked by the blue circle has a direction of 0 degrees. Thus, its gradient magnitude of 2 is filled into the bin of 0 degrees. The pixel marked by the red circle has a direction of 25 degrees, which falls between the adjacent bins of 20 degrees and 40 degrees. In this case, its gradient magnitude of 4 is distributed to these two bins based on linear distance weighting. Since 25 degrees is closer to 20 degrees, a larger portion is assigned to the bin representing 20 degrees. After all pixels’ gradients within the characteristic neighborhood are assigned, the final values of the nine bins are computed as the ratio of the accumulated gradient value in each bin to the overall sum of gradient values across all bins.

(11) $$b_{i}=\frac{G_{i}}{\sum_{i=1}^{9}G_{i}}$$

where $G_{i}$ Is the gradient value assigned to the *i*-th bin; $b_{i}$ is the final value of the *i*-th bin.

Figure 5b shows the gradient histogram of the first candidate target in Figure 4c. Figure 5c is the visual result of HOG. In Figure 5c, each line segment represents the bin whose direction is perpendicular to the line segment, and the length of the line segment represents the bin’s value. Visual HOG can intuitively display the gradient distribution of candidate targets.

Then, the GHEM of candidate targets is defined as:

(12) $$\mathrm{GHEM}={\left(1-\frac{\sigma_{b}}{\sigma_{\max}}\right)}^{2},$$

where $\sigma_{b}$ is the standard deviation of the nine bins; $\sigma_{\max}$ is the possible maximum value of $\sigma_{b}$; and the square in the formula is to enhance the differentiation. The value of $\sigma_{\max}$ are the solution of the following nonlinear programming problem.

(13) $$\begin{array}{c} \min f\left(x\right)=-\sqrt{\frac{1}{8}\sum_{i=1}^{9}{\left(b_{i}-{\bar{b}}_{i}\right)}^{2}}\\ s.t.\left\{\begin{array}{c} 0\leq b_{i}\leq 1\\ \sum_{i=1}^{9}b_{i}=1 \end{array}\right. \end{array}$$

where ${\bar{b}}_{i}$ is the average value of $b_{i}$. The practical meaning of this problem is to find the maximum standard deviation in the range of $b_{i}$. We can obtain that $\sigma_{\max}$ is equal to 0.3333 by solving this problem. If a candidate target is ideally isotropic, all bins will be equal, its standard deviation will be 0 and its GHEM will be 1. If one bin of a candidate target is 1 and the other bins are 0, such as a step edge region, its standard deviation is 0.3333 and its GHEM is 0.

**Figure 6:**
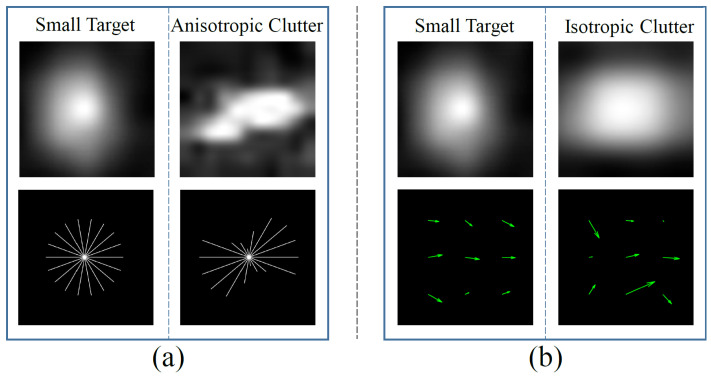
Comparison of HOG and optical flow between small target and sea clutter. (a) Visual HOG of a small target and an anisotropic clutter. (b) Visual optical flow vectors of a small target and an isotropic clutter. The green arrows represent the optical flow vectors.

Figure 6a shows two characteristic neighborhoods containing a small target and an anisotropic clutter, respectively, along with their visual HOG. In the visual HOG of the small target, all bins are close, indicating a balanced distribution of the gradients in different directions. The balanced HOG is consistent with the isotropic appearance of the small target. On the other hand, in the visual HOG of the anisotropic clutter, several bins are notably longer than the others, indicating a concentration of gradients in specific directions. The GHEM of the small target and clutter in Figure 6a are 0.9570 and 0.3081, respectively. The small target has higher GHEM than the clutter, which reflects the effectiveness of GHEM in distinguishing between small targets and anisotropic clutter.

### 2.3. Local Optical Flow Consistency Measure (LOFCM)

The optical flow method is a technique used to estimate the motion information of pixels between consecutive frames. It is commonly applied in various fields, such as target tracking, motion analysis, and 3D reconstruction. Optical flow refers to the motion vectors of pixels. As discussed in Section 1.2, a small target has a stable appearance, which means that the relative positions of its different parts will remain fixed over a short period (similar to a rigid body). Therefore, all pixels of a small target will possess consistent motion vectors. Conversely, sea clutter demonstrates an unstable appearance, with its shape constantly changing. As a result, some pixels inevitably have different displacements from others. Therefore, the consistency of the optical flow vectors of a candidate target can be used to characterize its appearance stability. In this regard, we propose the local optical flow consistency measure (LOFCM) based on the Lucas–Kanade (L-K) method.

The L-K method is a simple and classic optical flow algorithm [43]. The application of L-K method requires the following three basic conditions:

(1) Brightness constancy: The gray value of a pixel does not change over time.
(2) Small motion: The displacement of a pixel is small, and the passage of time cannot cause drastic changes in the pixel position.
(3) Local spatial consistency: The relative positions of neighboring pixels do not change.

Based on the above conditions, the optical flow calculation formula of the L-K method can be derived.

A(x,y,t) is used to represent the value of pixel (x,y) in the candidate target array of frame *t*. (Δx,Δy) is a displacement of the pixel in time Δt. According to Condition 1, the values of (x,y) before and after the displacement should be equal, so there are

(14) A(x,y,t)=A(x+Δx,y+Δy,t+Δt).

The right-hand side of the formula can be expanded in the Taylor series.

(15) $$A\left(x+\Delta x,y+\Delta y,t+\Delta t\right)=A\left(x,y,t\right)+\frac{\partial A}{\partial x}\Delta x+\frac{\partial A}{\partial y}\Delta y+\frac{\partial A}{\partial t}\Delta t+\varepsilon$$

where ε is a higher-order infinitesimal. According to Condition 2, the displacement (Δx,Δy) is small, so ε can be ignored, and we can obtain

(16) $$\frac{\partial A}{\partial x}\Delta x+\frac{\partial A}{\partial y}\Delta y+\frac{\partial A}{\partial t}\Delta t=0\Rightarrow \frac{\partial A}{\partial x}\frac{\Delta x}{\Delta t}+\frac{\partial A}{\partial y}\frac{\Delta y}{\Delta t}+\frac{\partial A}{\partial t}=0.$$

The above formula can be abbreviated as

(17) $$A_{x}v_{x}+A_{y}v_{y}+A_{t}=0,$$

where $A_{x}$, $A_{y}$ and $A_{t}$ are the partial derivatives of the candidate target array concerning *x*, *y*, and *t*; $\left(v_{x},v_{y}\right)$ is the optical flow vector. Based on Condition 3, we can assume that the pixels in a window have the same optical flow. Then we have

(18) $$\left[\begin{array}{cc} \begin{array}{c} A_{x1}\\ A_{x2}\\ \vdots \\ A_{xn} \end{array} & \begin{array}{c} A_{y1}\\ A_{y2}\\ \vdots \\ A_{yn} \end{array} \end{array}\right]\left[\begin{array}{c} v_{x}\\ v_{y} \end{array}\right]=\left[\begin{array}{c} -A_{t1}\\ -A_{t2}\\ \vdots \\ -A_{tn} \end{array}\right],$$

where the *n* in the superscript is the number of pixels in the window. Equation (18) can be abbreviated as

(19) $$K\vec{v}=-b,$$

where *K* is the matrix composed of $\left[A_{xi},\;A_{yi}\right]$; *b* is the vector composed of $A_{ti}$. The optical flow vector can be solved by the least square method.

(20) $$\vec{v}={\left(K^{T}K\right)}^{-1}K^{T}\left(-b\right)$$

After combining the items, Equation (20) can be expressed as

(21) $$\vec{v}=\left[\begin{array}{c} v_{x}\\ v_{y} \end{array}\right]={\left[\begin{array}{cc} \sum_{i=1}^{n}A_{xi}^{2} & \sum_{i=1}^{n}A_{xi}A_{yi}\\ \sum_{i=1}^{n}A_{xi}A_{yi} & \sum_{i=1}^{n}A_{yi}^{2} \end{array}\right]}^{-1}\left[\begin{array}{c} -\sum_{i=1}^{n}A_{xi}A_{ti}\\ -\sum_{i=1}^{n}A_{yi}A_{ti} \end{array}\right].$$

A(t) is used to represent the candidate target array of frame *t*, and the local regions of each candidate target in the Top-Hat transformation of frame t−1 and frame t+1 are also scaled and stitched to form two array images, represented by A(t−1) and A(t+1), respectively. The time domain gradient is calculated as follows.

(22) $$A_{t}^{-}=A\left(t\right)-A\left(t-1\right)$$

(23) $$A_{t}^{+}=A\left(t+1\right)-A\left(t\right)$$

where $A_{t}^{-}$ and $A_{t}^{+}$ are the time domain gradients along the negative and positive directions of the time axis, respectively. $A_{x}$ and $A_{y}$ can be calculated using Equations (7) and (8). Substitute $A_{x}$, $A_{y}$, $A_{t}^{-}$, and $A_{t}^{+}$ into Equation (21), and we can obtain

(24) $${\vec{v}}^{-}={\left[\begin{array}{cc} \sum_{i=1}^{n}A_{xi}^{2} & \sum_{i=1}^{n}A_{xi}A_{yi}\\ \sum_{i=1}^{n}A_{xi}A_{yi} & \sum_{i=1}^{n}A_{yi}^{2} \end{array}\right]}^{-1}\left[\begin{array}{c} -\sum_{i=1}^{n}A_{xi}A_{ti}^{-}\\ -\sum_{i=1}^{n}A_{yi}A_{ti}^{-} \end{array}\right],$$

(25) $${\vec{v}}^{+}={\left[\begin{array}{cc} \sum_{i=1}^{n}A_{xi}^{2} & \sum_{i=1}^{n}A_{xi}A_{yi}\\ \sum_{i=1}^{n}A_{xi}A_{yi} & \sum_{i=1}^{n}A_{yi}^{2} \end{array}\right]}^{-1}\left[\begin{array}{c} -\sum_{i=1}^{n}A_{xi}A_{ti}^{+}\\ -\sum_{i=1}^{n}A_{yi}A_{ti}^{+} \end{array}\right],$$

where ${\vec{v}}^{-}$ and ${\vec{v}}^{+}$ are the optical flow vectors on both sides of A(n) in the time domain. The final optical flow vector is the mean of ${\vec{v}}^{-}$ and ${\vec{v}}^{+}$.

(26) $$\vec{v}=\frac{1}{2}\left({\vec{v}}^{-}+{\vec{v}}^{+}\right)$$

**Figure 7:**
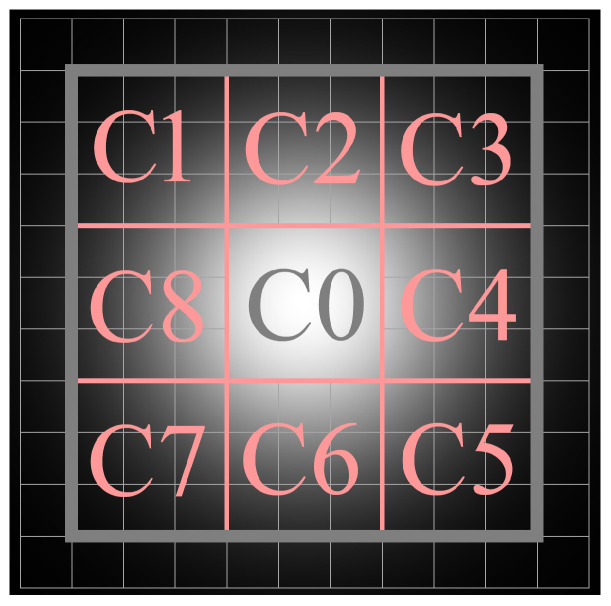
Schematic diagram of characteristic neighborhood division.

Each characteristic neighborhood is divided as shown in Figure 7. C0 is the 9 × 9 window within the characteristic neighborhood. The optical flow vector of C0 is represented by ${\vec{v}}_{0}$, which can be calculated using Equations (22)–(26). The number of pixels involved in the calculation, namely *n* in Equation (21), is equal to 81. C0 can be further subdivided into cells of 3 × 3, each cell containing 9 pixels. The surrounding eight cells are labeled as C1 to C8. Similarly, the optical flow vectors of these cells, denoted as ${\vec{v}}_{0}$–${\vec{v}}_{8}$, can also be calculated using Equations (22)–(26). Here, each cell employs 9 pixels for the calculation. Figure 4e shows the optical flow vectors of the candidate targets in Figure 4c. To enhance the visual effect, all optical flow vectors have been magnified threefold in size.

The LOFCM of candidate targets is defined as:

(27) $$\mathrm{LOFCM}=1-\sqrt{\frac{\sum_{i=1}^{8}{\left({\vec{v}}_{i}-{\vec{v}}_{0}\right)}^{2}}{8{\vec{v}}_{0}^{2}+\varepsilon }}.$$

where ε is set to 0.001 to prevent the denominator from being 0.

It should be noted that Condition 3 of the L-K method requires that the relative positions of adjacent pixels do not change, which means that the adjacent pixels must maintain consistent motion. In the calculation of the optical flow vectors, Equation (18) is built based on Condition 3. When all the pixels involved in Equation (18) have the same displacement, the ${\left[v_{x},\;v_{y}\right]}^{T}$ calculated by solving Equation (18) will represent the true displacement for each pixel. However, as previously discussed, the shape change of sea clutter will cause certain pixels to move inconsistently with others, so sea clutter does not satisfy Condition 3. For sea clutter pixels, the pixels involved in Equation (18) may have different displacements, so the calculated ${\left[v_{x},\;v_{y}\right]}^{T}$ is not the true displacement of each pixel. In this case, the result of solving Equation (18) by the least square method represents the average motion trend of these pixels, which is equivalent to the displacement of the cell. As can be seen from Equation (27), the calculation of LOFCM only relies on the displacements of the nine cells, and it does not require exact displacement values for every pixel. Therefore, although sea clutter does not meet Condition 3 of the L-K method, the calculation process of LOFCM described in Equations (22)–(27) is still applicable to all candidate targets.

In the characteristic neighborhood of the candidate target, the window C0 encompasses all the pixels belonging to the candidate target. The optical flow vector ${\vec{v}}_{0}$ reflects the overall movement trend of these pixels and can be considered as the actual displacement of the candidate target. Each cell within the characteristic neighborhood only contains a subset of pixels located on the edges of the candidate target. As a result, the optical flow vectors ${\vec{v}}_{0}$–${\vec{v}}_{8}$ can only reflect the movement trend of a single part of the candidate target. The difference between ${\vec{v}}_{0}$–${\vec{v}}_{8}$ and ${\vec{v}}_{0}$ indicates the relative displacement of the individual parts to the whole target, namely the deformation. If the candidate target remains entirely undeformed, ${\vec{v}}_{0}$–${\vec{v}}_{8}$ will equal ${\vec{v}}_{0}$, resulting in a LOFCM value of 1. However, if the candidate target exhibits deformation, its LOFCM will be less than 1. If the numerator in Equation (27) exceeds the denominator, signifying that the average deformation vector of the eight cells exceeds the total displacement, the LOFCM value will be less than 0.

Figure 6b shows the characteristic neighborhoods of a small target and an isotropic clutter, along with their optical flow vectors. On the whole, the optical flow vectors of the small target have a similar direction, indicating that the stable appearance ensures all parts of the small target keep moving consistently. In contrast, the optical flow vectors of the clutter lack consistency, which is attributed to its ongoing deformation process. Although the current appearance of the clutter is isotropic, it cannot be maintained indefinitely. Over time, the clutter will transition from isotropic to anisotropic, thereby exhibiting differences from the small target. However, we can distinguish between the small target and the isotropic clutter in the current moment through optical flow. In essence, LOFCM converts the spatial features that would only appear in the future into spatial–temporal features that are exploitable in the present moment. In Figure 6b, the LOFCM values of the small target and the clutter are 0.7540 and 0.0235, respectively. The small target has a higher LOFCM value than that of the clutter, reflecting the effectiveness of LOFCM in distinguishing between small targets and isotropic clutter.

### 2.4. Appearance Stable Isotropy Measure

The location mapping function in Equation (6) is used to map the GHEM and LOFCM of each characteristic neighborhood back to the original image. GHEM and LOFCM play a major role in distinguishing anisotropic and isotropic clutter, respectively, so we fuse GHEM and LOFCM as weighting coefficients for Top-Hat images. Finally, ASIM is defined as

(28) $$\mathrm{ASIM}=\left\{\begin{array}{cc} T\times \mathrm{GHEM}\times \mathrm{LOFCM} & \mathrm{GHEM}>0.5,\mathrm{LOFCM}>0.1\\ 0 & \mathrm{else} \end{array}\right..$$

The classification conditions in the above formula are derived from statistical experience during the development process. Generally, the GHEM of a small target will not be lower than 0.5, and its LOFCM will not be lower than 0.1. The calculation process of ASIM is shown in Algorithm 1.

Small target has high GHEM and LOFCM, so it will be retained in ASIM. Sea clutter is difficult to have both high GHEM and LOFCM, so it will be suppressed in ASIM. Figure 4f shows the ASIM of Figure 4a, and most of the sea clutter has been suppressed. Compared with the original image with a heavy sea clutter environment, ASIM is more conducive to extracting small targets. In the subsequent processing, we can extract the target by a simple threshold segmentation.

(29) $$TH_{\mathrm{ASIM}}=\mu_{\mathrm{ASIM}}+k_{\mathrm{ASIM}}\times \sigma_{\mathrm{ASIM}}$$

where $TH_{\mathrm{ASIM}}$ is the segmentation threshold of ASIM; $\mu_{\mathrm{ASIM}}$ and $\sigma_{\mathrm{ASIM}}$ are the mean and standard deviation of ASIM, respectively; and $k_{\mathrm{ASIM}}$ is the coefficient. The value of $k_{\mathrm{ASIM}}$ will determine the threshold level. Increasing $k_{\mathrm{ASIM}}$ will lead to a decrease in both the false alarm rate and detection rate, while decreasing $k_{\mathrm{ASIM}}$ will result in an increase in both of them. It should be noted that different tasks prioritize either the detection rate or the false alarm rate depending on their specific requirements. For example, in military defense applications, achieving a high detection rate is typically of utmost importance due to the potential catastrophic consequences of missing the target. In the civil field, the false alarm rate is usually required to be at a low level to save resources and time costs. This paper adopts the detection rate priority strategy. According to the development experience, $k_{\mathrm{ASIM}}$ is set as 0.5, which can ensure that no target is missed on all experimental sequences.
**Algorithm 1** ASIM.**Input:** frame t−1, *t*, and t+1**Output:** ASIM image
1:Calculate the Top-Hat transformation of the input images using Equations (1) and (2), where the Top-Hat image of frame *t* is represented by *T*;2:Extract candidate targets from *T* according to Equations (3)–(5);3:The characteristic neighborhoods of the candidate targets in the Top-Hat transformation of the input images are scaled and stitched to obtain the array images A(t−1), A(t), and A(t+1);4:Calculate *G* and θ of the pixels in A(t) according to Equations (7)–(10);5:Calculate the oriented gradient histogram of each candidate target according to Figure 5a and Equation (11);6:$\mathrm{GHEM}={\left(1-\frac{\sigma_{b}}{\sigma_{\max}}\right)}^{2}$;7:Calculate the the optical flow vectors ${\vec{v}}_{0}$–${\vec{v}}_{8}$ of each candidate target in A(t) according to Equations (20)–(24);8:$\mathrm{LOFCM}=1-\sqrt{\frac{\sum_{i=1}^{8}{\left({\vec{v}}_{i}-{\vec{v}}_{0}\right)}^{2}}{8{\vec{v}}_{0}^{2}}}$;9:$\mathrm{ASIM}=\left\{\begin{array}{cc} T\times \mathrm{GHEM}\times \mathrm{LOFCM} & \mathrm{GHEM}>0.5,\mathrm{LOFCM}>0.1\\ 0 & \mathrm{else} \end{array}\right..$


## 3. Experiments

**Figure 8:**
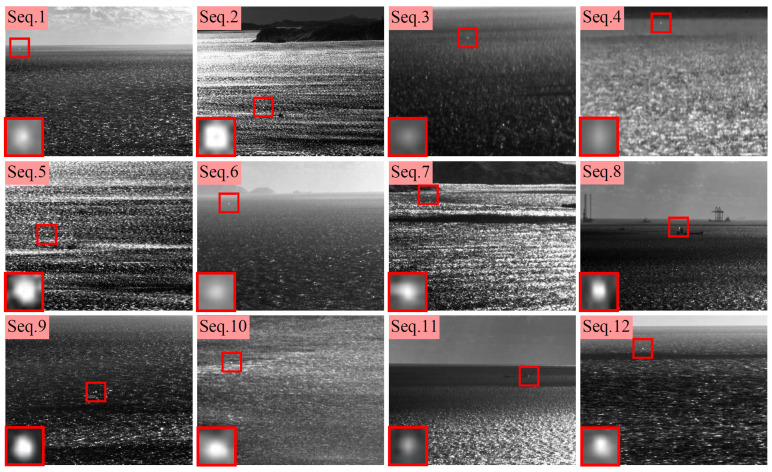
First frame of the experimental sequences. The small targets are marked with red boxes, and their enlarged views are placed in the left-bottom corner of the images.

This paper organized experiments to evaluate the performance of the proposed method. There are 12 sequences selected as datasets, denoted as Seq.1–Seq.12, respectively. Figure 8 shows the first frame of each sequence. In Figure 8, all small targets are marked with a red box, and their enlarged views are placed in the bottom left corner of the images. The background of these sequences is the sea surface with a considerable amount of clutter. Detailed information of the datasets is provided in Table 1. To objectively measure the performance of the proposed method, nine classic or advanced detection methods are selected as baseline methods, including LCM [15], IPI [26], TLLCM [19], WSLCM [21], NOLC [29], ADMD [14], STLCF [22], STLCM [23], and MSL-STIPT [35]. The experiments were conducted on a computer with an Intel Core i7-6700 CPU (Intel, Santa Clara, CA, USA) and 16 GB of memory, and the relevant code was implemented on MATLAB 2016a.

### 3.1. Evaluation Metrics

In this paper, the signal-to-clutter ratio (SCR) gain (SCRG), background suppression factor (BSF), and receiver operating characteristic (ROC) curves are used as evaluation indexes. BSF and SCRG measure the background suppression and target enhancement capabilities of detection methods, respectively [11]. BSF, SCR, and SCRG are defined as

(30) $$\mathrm{BSF}=\frac{\sigma_{\mathrm{in}}}{\sigma_{\mathrm{out}}+\varepsilon },\;\mathrm{SCR}=\left|\frac{I_{T}-\mu_{B}}{\sigma_{B}}\right|,\;\mathrm{SCRG}=\left|\frac{\mathrm{SC}R_{\mathrm{out}}}{\mathrm{SC}R_{\mathrm{in}}+\varepsilon }\right|,$$

where $\sigma_{\mathrm{in}}$ and $\sigma_{\mathrm{out}}$ are the standard deviations of the input image and the output image, respectively. $I_{T}$ is the intensity of the target; $\mu_{B}$ and $\sigma_{B}$ are the mean and standard deviation of the target neighborhood pixels (excluding the target pixels), respectively; $\mathrm{SC}R_{\mathrm{in}}$ and $\mathrm{SC}R_{\mathrm{out}}$ are the SCR of the input image and output image, respectively. ε is set to 0.001 to prevent the denominator from being 0.

ROC curve describes the relationship between the detection rate (DR) and the false alarm rate (FAR). A high ROC curve indicates that the detection method can achieve a high detection rate under the same false alarm rate. DR and FAR are defined as

(31) $$\mathrm{DR}=\frac{\mathrm{number}\;\mathrm{of}\;\mathrm{detected}\;\mathrm{true}\;\mathrm{targets}}{\mathrm{total}\;\mathrm{number}\;\mathrm{of}\;\mathrm{true}\;\mathrm{targets}},$$

(32) $$\mathrm{FAR}=\frac{\mathrm{number}\;\mathrm{of}\;\mathrm{pixels}\;\mathrm{in}\;\mathrm{false}\;\mathrm{alarms}}{\mathrm{total}\;\mathrm{number}\;\mathrm{of}\;\mathrm{pixels}\;\mathrm{in}\;\mathrm{the}\;\mathrm{whole}\;\mathrm{image}}.$$

### 3.2. Experimental Results

**Figure 9:**
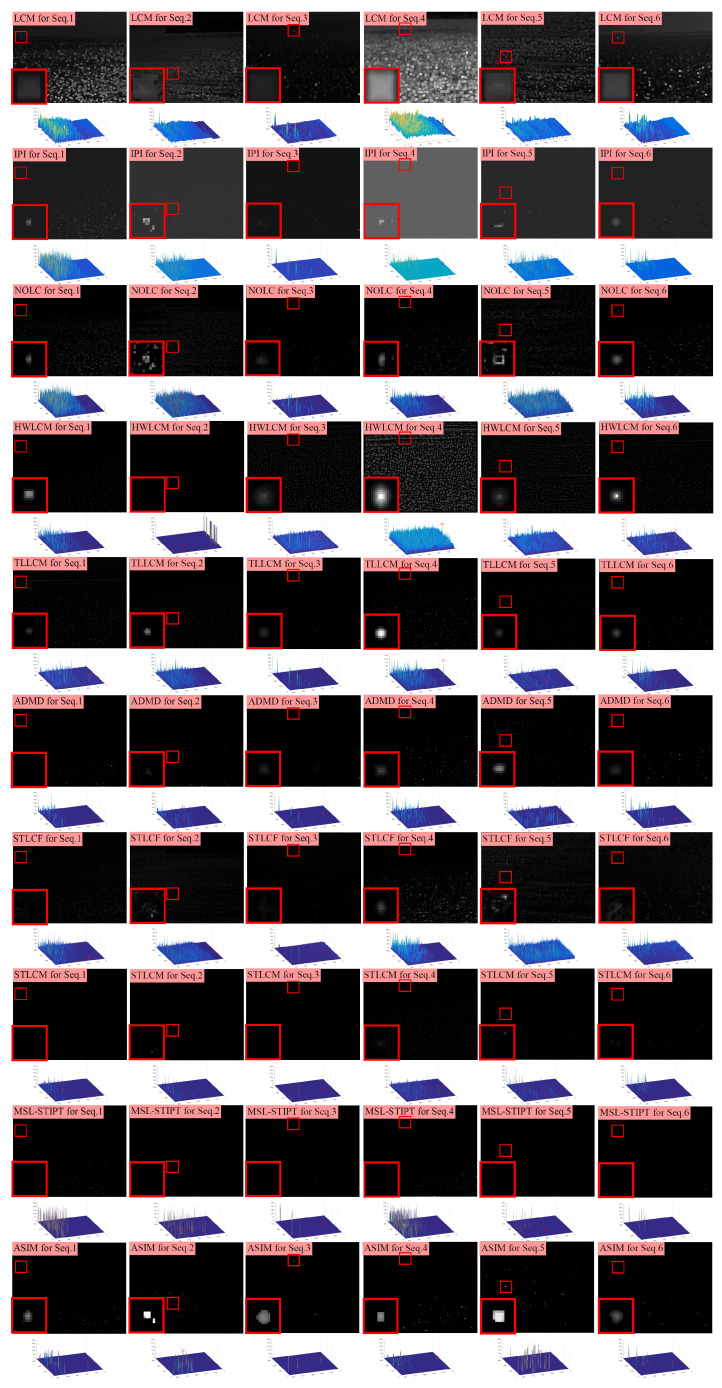
The resulting images of different methods on Seq.1–6. The target area in each resulting image is marked with a red box and its enlarged view is placed in the left-bottom corner.

**Figure 10:**
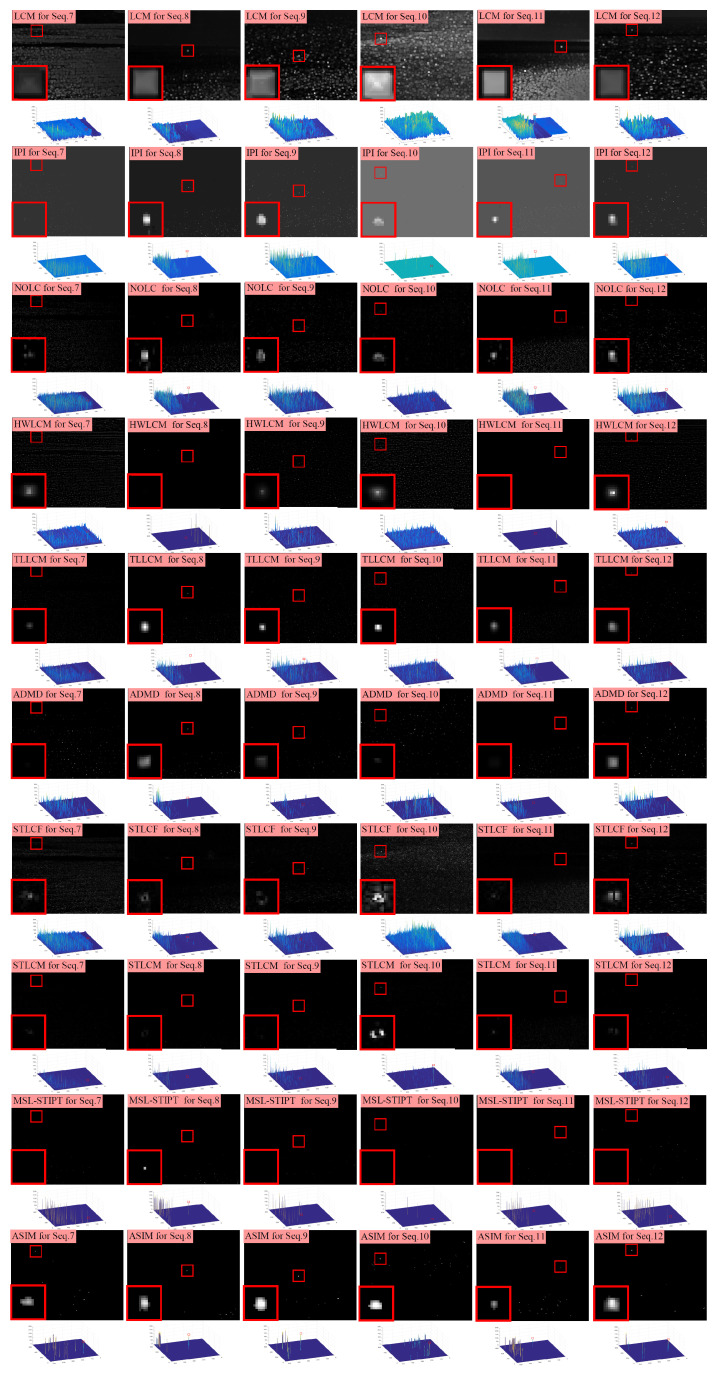
The resulting images of different methods on Seq.7–12. The target area in each resulting image is marked with a red box and its enlarged view is placed in the left-bottom corner.

First, Figure 9 and Figure 10 show the processing results of different methods on the 12 experimental sequences, along with their corresponding 3D surface plots. In the result images, the target positions are marked with red rectangles, while zoomed-in views of these marked regions are presented in the bottom left corner. In the 3D surface plots, peaks belonging to the targets are also marked with red rectangles. These processing results can intuitively demonstrate the ability of different methods in background suppression and target enhancement.

LCM, IPI, NOLC, HWLCM, TLLCM, ADMD, and STLCF can enhance the targets to some extent; however, their effectiveness in suppressing clutter is limited. There are a large number of peaks in their 3D plots, which correspond to residual clutter. STLCM and MSL-STIPT exhibit better background suppression effects with fewer residual clutter peaks observed in their 3D plots. However, both STLCM and MSL-STIPT mistakenly suppressed the real targets in many sequences. Among all the methods, the proposed method in this paper shows the best overall performance in terms of background suppression and target enhancement. Our method can effectively enhance the targets while achieving relatively little clutter residue.

To quantitatively compare the background suppression and target enhancement abilities of different methods, Table 2 provides their BSF and SCRG. Each value in Table 2 is the average calculated over all images within the corresponding sequence. The first to third places for each sequence are highlighted in bold red, blue, and green, respectively. Among the baseline methods, excluding STLCM and MSL-STIPT, the BSF values are generally low, with most not exceeding 20. Although STLCM and MSL-STIPT perform better than other baseline methods in terms of BSF, they still fall short compared to our proposed method. LCM, HWLCM, TLLCM, STCLF, and STLCM have low SCRG across all sequences. IPI, NOLC, ADMD, and MSL-STIPT have very high SCRG on some sequences but very low SCRG on others, indicating unstable performance across different sequences. The proposed method achieves the highest BSF on Seq.1, 2, 3, 4, 6, 7, 8, 9, and 12, surpassing the second-ranked method by 10.92%, 25.65%, 3.29%, 1.55%, 18.88%, 22.77%, 13.81%, and 23.67%, respectively. In terms of SCRG, our proposed method has the highest values for Seq.1–10, surpassing the second-ranked method by 28.84%, 426,692.00%, 296.98%, 130.74%, 191.13%, 90.54%, 3053.18%, 2881.76%, 28.79%, and 82,512.27%, respectively. Additionally, the BSF and SCRG values of our method rank among the top three on all sequences, demonstrating its superior performance in terms of both background suppression and target enhancement.

The performance of different methods in terms of SCRG and BSF can be attributed to their underlying principles. LCM, HWLCM, TLLCM, and ADMD are methods based on central-surround differences, while they can enhance small targets to some extent, they struggle with suppressing sea clutter, which also exhibits high local contrast. Among these methods, ADMD performs relatively better in SCRG. This is because ADMD zeros out the pixels in the target neighborhood during computation, which can significantly improve the SCR of the target when there is no other strong clutter within the target neighborhood (e.g., Seq3, 4, 6, 8, and 9). IPI and NOLC are based on the sparsity model of small targets. They can improve the target SCR by effectively separating the small targets from the background. However, they show poor background suppression effects as many instances of sea clutter also satisfy the assumption of local sparsity, leading to mistaken extraction of clutter as targets. STLCF and STLCM utilize spatiotemporal filtering, primarily measuring the saliency of local regions in spatial and temporal domains. However, since both small targets and sea clutter are bright and moving, they generate strong spatiotemporal saliency for both. STLCM imposes shape constraints on salient regions, thus performing slightly better overall than STLCF. MSL-STIPT is based on the modified spatial–temporal tensor model, wherein the constraints are overly strict, making it easy to suppress both targets and clutter simultaneously. In contrast, our proposed method takes full advantage of the distinctions between small targets and clutter in spatiotemporal features, thereby achieving superior overall performance in target enhancement and background suppression compared to baseline methods.

**Figure 11:**
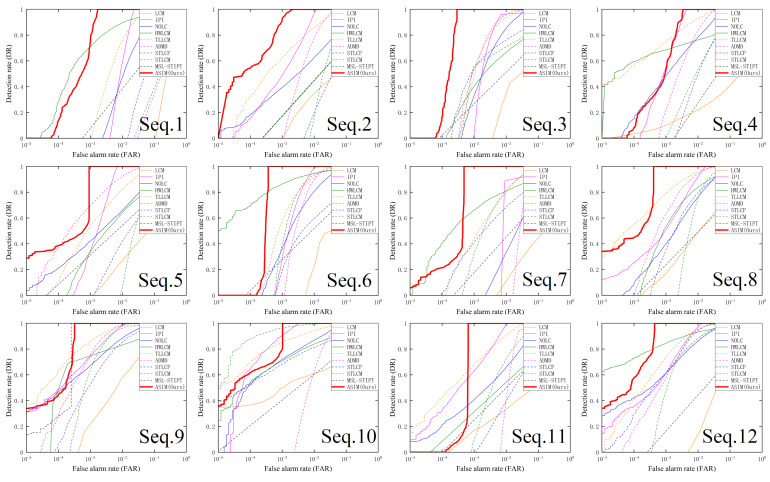
Receiver operating characteristic (ROC) curves of different methods.

Secondly, to evaluate the comprehensive detection performance of different methods, this paper presents their ROC curves, as shown in Figure 11. These ROC curves are obtained by adjusting the segmentation threshold from high to low, while the ROC curve of our method may initially be lower than some other methods in the high threshold stage (e.g., on Seq. 1 compared to HWLCM), it should be noted that very high thresholds are typically not employed in practice since most detection tasks prioritize a high detection rate. At the low threshold stage, our method exhibits significant advantages over the baseline methods. Table 3 shows the detection rates of different methods when the false alarm rate is $10^{-3}$, and the highest detection rate on each sequence is marked in bold red. When the false alarm rate is $10^{-3}$, the detection rate of our method is the highest on most sequences and reached 1 on Seq.3, 5, 6, 7, 8, 9, 11, and 12. On Seq.4, although the detection rate of our method is temporarily lower than that of TLLCM and HWLCM, it can be seen from the ROC curves of Seq.4 that the detection rate of our method is still the first to reach 1. The above results show that our method can achieve a higher detection rate with the same false alarm rate compared to the baseline methods.

Table 4 shows the false alarm rates of different methods at a detection rate of 1, with the lowest false alarm rate marked in bold red for each sequence. Our method has the lowest false alarm rate across all sequences, except for Seq.9. On Seq.9, while MSL-STIPT has a slightly lower false alarm rate, the difference is not significant compared to our method. Furthermore, MSL-STIPT demonstrates poor performance on other sequences. These results show that our method can achieve a lower false alarm rate at the same detection rate. The analysis of the ROC curve, along with Table 3 and Table 4, leads to the conclusion that our method exhibits superior detection performance compared to the baseline methods.

Finally, Table 5 provides the average computational time for different methods on Seq.1. Generally, methods based on spatial or spatiotemporal filtering exhibit low computational time, such as LCM, HWLCM, ADMD, STLCF, and STLCM. Although TLLCM is also a spatial filtering method, its computation involves numerous sorting operations, resulting in a higher time consumption. Methods based on optimization, including IPI, NOLC, and MSL-STIPT, typically demonstrate high computational overhead due to iterative operations. The heavy computational burden limits their practical application. Our method ranks fifth among all methods. Although LCM, ADMD, STLCF, and STLCM are faster than our method, they cannot achieve both fast speed and good detection performance simultaneously. Considering the comprehensive performance of our method in terms of BSF, SCRG, ROC curves, and computational time, it can be concluded that our method outperforms the baseline methods in heavy sea clutter environments.

## 4. Conclusions

This paper analyzes the differences in appearance between IR small targets and sea clutter in spatial and temporal domains. Based on this analysis, a detection method utilizing the appearance stable isotropy measure (ASIM) is proposed. First, the Top-Hat transform is employed to extract the salient regions and generate the candidate target array image. Then, two novel features, namely GHEM and LOFCM, are proposed to characterize the isotropy and stability of the candidate targets’ appearance, respectively. Finally, this paper fuses GHEM and LOFCM and uses threshold segmentation to determine the final target. Experiments are conducted on sequences with heavy sea clutter environments to evaluate the performance of the proposed method. The experimental results show that the proposed method performs better than the baseline methods in comprehensive detection performance. 

## Figures and Tables

**Table 1:** Details of the experimental sequences.

Sequence	Image Size	Frame Number	Target Type	Target Size
Seq.1	512 × 640	1000	Signal light	5 × 4
Seq.2	512 × 640	1000	Signal light	3 × 3
Seq.3	512 × 640	1000	Ship	9 × 9
Seq.4	512 × 640	97	Ship	9 × 7
Seq.5	512 × 640	400	Signal light	5 × 5
Seq.6	512 × 640	500	Ship	7 × 7
Seq.7	512 × 640	1000	Ship	5 × 5
Seq.8	512 × 640	1000	Signal light	7 × 7
Seq.9	512 × 640	1000	Signal light	6 × 5
Seq.10	512 × 640	500	Ship	5 × 6
Seq.11	512 × 640	500	Signal light	4 × 4
Seq.12	512 × 640	500	Ship	6 × 6

**Table 2:** Background suppression factor (BSF) and signal-to-clutter ratio gain (SCRG) of different methods.

	Sequence	LCM	IPI	NOLC	HWLCM	TLLCM	ADMD	STLCF	STLCM	MSL-STIPT	ASIM (Ours)
BSF	Seq.1	2.35	5.9603	4.6786	11.5765	13.2104	16.4142	13.1323	17.1572	14.2337	**19.0300**
	Seq.2	4.3029	11.4957	3.9490	18.1301	12.8368	14.6817	7.9277	12.5154	22.4095	**28.1576**
	Seq.3	4.4258	15.3305	11.1006	4.7119	14.8072	16.3769	19.2110	**32.4433**	19.3694	25.6798
	Seq.4	0.9816	10.8962	5.2172	1.5451	5.2782	7.6194	3.6652	7.3866	5.2973	**11.2543**
	Seq.5	4.2431	13.1191	3.6601	6.6224	19.0589	14.3481	5.2402	26.9579	**36.2472**	20.5585
	Seq.6	4.0975	18.4130	9.9744	11.6162	19.1893	16.3994	10.5474	34.6185	32.4306	**35.1543**
	Seq.7	3.3625	12.1870	4.4644	6.9145	13.1137	11.9082	5.0353	17.9317	15.4038	**21.3181**
	Seq.8	1.5828	3.9114	3.4861	6.4551	6.6746	13.0041	6.3620	15.2050	14.8925	**18.6672**
	Seq.9	4.3457	9.3311	5.9340	26.0391	12.5617	13.7088	11.3957	31.7395	15.6627	**36.1225**
	Seq.10	1.1671	20.0762	6.2541	3.1570	5.4764	5.2878	1.7990	16.1800	**40.0401**	17.3463
	Seq.11	1.1776	8.1043	2.7801	**46.4676**	9.3482	11.5604	4.5328	10.0845	14.8832	18.8061
	Seq.12	2.9323	8.6763	5.9026	8.5013	14.5613	10.3476	8.5029	19.9019	17.4160	**24.6129**
SCRG	Seq.1	1.3695	11,297.0	15,147.0	5.0528	20.1239	0.7885	1.7908	11.7536	0.0028	**19,515.0**
	Seq.2	0.7327	6.6738	1.9079	2.9436	3.0579	16.5467	2.9956	5.3132	0.00097	**70,620.0**
	Seq.3	1.2000	4.7764	2.9948	1.2734	4.1211	2929.9	0.7823	7.6465	0.0082	**11,631.0**
	Seq.4	0.9025	2.0318	1.8720	1.6600	103.5336	4017.0	33.9769	29.6237	0.00034	**9268.8**
	Seq.5	1.3870	19,649.0	3.9910	2.5819	6.7425	683.2696	4.5386	18.0894	0.0044	**57,204.0**
	Seq.6	2.7673	3924.2	21.6796	9.2779	59.9930	2897.8	0.6596	50.1290	0.9172	**7477.0**
	Seq.7	0.9780	1756.7	3.0760	4.6140	9.5489	1.6425	5.4671	20.5076	0.2858	**55,392.0**
	Seq.8	0.7010	1.1465	1.2271	1.4607	2.5313	1315.8	1.0593	0.2334	0.7537	**39,234.0**
	Seq.9	3.5557	9.0137	2.9227	5.1908	32.2832	12,069.0	3.0763	8.4399	16,002.0	**20,609.0**
	Seq.10	1.5822	83.3607	5.7312	4.9129	12.4931	53.2729	4.8203	95.2062	0.5678	**78,652.0**
	Seq.11	2.5551	**8366.6**	3.3482	5.2843	48.1007	570.8405	2.4935	43.9950	0.0759	7859.3
	Seq.12	2.3255	18.5972	3.5414	5.9537	6.7523	**9164.9**	2.9079	14.6723	0.0540	7665.2

The red bold, blue, and green numbers represent the first, second, and third place on each sequence, respectively.

**Table 3:** Detection rates of different methods when the false alarm rate is $10^{-3}$.

Sequence	LCM	IPI	NOLC	HWLCM	TLLCM	ADMD	STLCF	STLCM	MSL-STIPT	ASIM (Ours)
Seq.1	0	0	0	0.7150	0	0	0	0	0.0739	**0.7475**
Seq.2	0	0.4943	0.3973	0.1650	0.6295	0	0	0	0.1684	**0.9201**
Seq.3	0	0.0724	0.3452	0.4049	0.5003	0.5415	0.5607	0.6622	0.2714	**1**
Seq.4	0.1029	0.5633	0.4900	0.6892	**0.7477**	0.2229	0	0.0316	0	0.5379
Seq.5	0	0.3437	0.4234	0.3393	0.5350	0.6957	0	0	0.3231	**1**
Seq.6	0	0.2956	0.3626	0.8668	0.5800	0	0.1935	0.4197	0.2814	**1**
Seq.7	0	0	0	0.6627	0.4506	0	0.4121	0.3474	0.1681	**1**
Seq.8	0.1716	0.5123	0.4016	0.5342	0.7925	0.6323	0.3535	0	0.2359	**1**
Seq.9	0.2210	0.7423	0.6756	**1**	0.8600	0.7581	0.5324	0.6448	**1**	**1**
Seq.10	0.5016	0.9114	0.7225	0.7100	0.8574	0	0.6861	0.9682	0.4055	**0.9951**
Seq.11	0.1773	0.6337	0.4169	0.3200	0.6825	0	0	0.0966	0.2575	**1**
Seq.12	0	0.6149	0.6130	0.8457	0.7375	0.5780	0.6130	0.4835	0.1723	**1**

The red bold number represents the maximum value on each sequence.

**Table 4:** False alarm rates of different methods when the detection rate is 1.

Sequence	LCM	IPI	NOLC	HWLCM	TLLCM	ADMD	STLCF	STLCM	MSL-STIPT	ASIM (Ours)
Seq.1	0.9917	0.0227	0.1174	0.3823	0.0618	1	0.6471	1	1	**0.0017**
Seq.2	0.9602	0.0127	0.1560	1	0.0762	0.0377	0.6531	0.0608	1	**0.0021**
Seq.3	0.9997	0.0069	0.0436	0.7130	0.0312	0.0178	0.4440	0.2949	1	**0.0003**
Seq.4	1	0.0044	0.0687	0.7914	0.0371	0.0387	0.4233	0.1480	1	**0.0035**
Seq.5	0.9995	0.0072	0.1552	0.7100	0.0793	0.0573	0.8377	0.1011	1	**0.0009**
Seq.6	1	0.0105	0.0957	0.3781	0.0496	0.0264	0.8411	0.0161	1	**0.0004**
Seq.7	1	0.0091	0.1644	0.6112	0.0680	0.0512	0.7353	0.1015	1	**0.0005**
Seq.8	0.9912	0.0176	0.0791	0.3980	0.0439	0.0305	0.5918	0.0302	1	**0.0004**
Seq.9	0.9990	0.0131	0.0774	0.0004	0.0257	0.0143	0.4058	0.0137	**0.0003**	0.0004
Seq.10	1	0.0035	0.0749	0.6108	0.0694	0.0564	0.8647	0.0323	1	**0.0010**
Seq.11	1	0.0109	0.1268	1	0.0394	0.0255	0.6525	0.1461	1	**0.0007**
Seq.12	0.9992	0.0141	0.0850	0.3586	0.0407	0.0295	0.3816	0.0234	1	**0.0004**

The red bold number represents the minimum value on each sequence.

**Table 5:** Calculation time (seconds/frame) of different methods.

LCM	IPI	NOLC	HWLCM	TLLCM	ADMD	STLCF	STLCM	MSL-STIPT	ASIM (Ours)
0.1427	139.3701	1071.0000	3.0719	28.4633	0.0054	0.0135	0.0376	84.3324	2.6118

## Data Availability

Data are contained within the article.
